# The power of ionic movements in plants

**DOI:** 10.1111/nph.70807

**Published:** 2025-12-08

**Authors:** Rainer Hedrich, Ines Kreuzer

**Affiliations:** ^1^ Faculty of Synthetic Biology Shenzhen University of Advanced Technology No. 1 Gongchang Road, Guangming District 518107 Shenzhen China; ^2^ Institute of Emerging Agricultural Technology Shenzhen University of Advanced Technology No. 1 Gongchang Road, Guangming District 518107 Shenzhen China; ^3^ State Key Laboratory of Quantitative Synthetic Biology Shenzhen Institute of Synthetic Biology, Shenzhen Institutes of Advanced Technology, Chinese Academy of Sciences No. 1 Gongchang Road, Guangming District 518107 Shenzhen China; ^4^ Julius‐von‐Sachs‐Institute for Biosciences, Department of Molecular Plant Physiology and Biophysics University of Wuerzburg Julius‐von‐Sachs‐Platz 2 97082 Wuerzburg Germany

**Keywords:** *Dionaea muscipula*, optogenetics, plant action potential, plant calcium signaling, plant physiology

## Abstract

The movement of ion‐driven electrogenic events known as plant action potentials in the Venus flytrap *Dionaea muscipula* has first been recognized in Darwin's time. Besides electrophysiological techniques making use of current‐ and voltage‐recording electrodes, today an ever‐growing spectrum of tools has become available, that  report online changes in membrane potential and ion concentration. This represents a big step forward, particularly in comparison to the ‘dark’ times when calcium‐signaling studies could not take advantage of Ca^2+^ reporters. Very recently, the first tools from a potpourri of light‐gated ion channels routinely used in neurobiology took the plant signaling field to a new level. This kind of genetically encoded, noninvasive opto‐tools can be activated by light and provide for remote controlling the membrane potential and ionic second messengers such as Ca^2+^ and H^+^. In future studies, such optogenetic tools in combination with the appropriate reporters for ionic and electrical impulses will allow studying membrane‐delimited early steps in plant signal transduction. Moreover, this toolbox will help us tackle the question of how, for example, Ca^2+^ and/or electrical signatures are assessed in terms of local and long‐distance information management.

## Introduction

Imagine a salad bowl decorated with vegetables that – similar to the Venus flytrap – snaps shut when touched with a fork and knife. Most people would step away from eating any of the green stuff when realizing that fresh vegetables are alive indeed. If we shrank to the size of a fly or ant, we would find ourselves in a life‐threatening situation when coming into contact with the capture organ of carnivorous plants. Pollinators such as bumble bees will face the power of plant movement when they visit the monkeyflower: collision with the bilobed touch‐sensitive filaments of the stigma will induce its closure (Fetscher & Kohn, 1999) and prevent self‐pollination or secondary fertilization.

If one even shrank to the size of bacteria or fungal cells that colonize a plant's above‐ and below‐ground tissues, we would still be facing a different world. The pollen germinating on the stigma papillae grows a tube toward the unfertilized eggs in the ovary. While the pollen tube grows within the transmitting tract of the stigma, root hairs of legumes form an infection thread when they come into contact with their nitrogen‐fixing symbionts that guides these beneficial microbes toward their final site, that is, the nitrogen‐fixing nodule. Like pollen tube growth, that of root hairs belongs to the fastest movements of single plant cells. On the leaf surface, visitors will find entrance ports called stomata, which are formed by a guard cell pair that can open and close: the stomatal pore. Upon recognizing pathogenic microbes, stomata close and prevent access to the inside of the leaf.

In all these microscopic scenarios ions either carry information or serve to regulate the osmotic potential. For osmotic functions, large quantities (mass flow) of mostly K^+^ and accompanying anions need to be shuttled. The increase in osmotic pressure reinforces the turgor and in turn drives cell expansion. In contrast to osmotically driven cell expansion, polarizing the membrane potential only needs a small number of ions to move across the cell membrane. These electrical signals are well known to occur when plants interact with microbes or experience sudden changes in their abiotic environments. Such episodic membrane depolarizations are associated with a transient rise in the cytosolic second messengers Ca^2+^ and H^+^ and carry information between cells (short distance) as well as between tissues and organs (long distance).

This article will address the nature and role of ion‐based electrical events in the plant systems able to rapidly change size and/or shape. The principles and the biology of electric ion movement will be exemplified on the action potential (AP) of the Venus flytrap and compared to osmotic ion movement in stomatal guard cells. Besides the latter two well‐studied systems, we discuss the potential of the monkeyflower Mimulus stigma as an attractive new model for combined electro‐osmotic studies.

## The plant action potential is not a matter of plant size and organization

In organisms, as small as bacteria, one might not assume that in such a single‐cell biological system communication via action potentials could have presented a selective advantage in evolution. In algal cells reaching a size of up to 20 cm, for example, *Chara* internodal cells (Foissner & Wasteneys, 2014), the action potential may report the change in solute concentration/composition of the extracellular space or the metabolic state from one end to the other. Neurons feature voltage‐dependent Na^+^, Ca^2+^ and K^+^ channels and ionotropic glutamate receptors and orthologs of these transporters have also been identified in bacteria (Chen *et al*., 1999; LeMasurier *et al*., 2001; Ren *et al*., 2001; Catterall & Zheng, 2015; Shimomura *et al*., 2020). Furthermore, electrical signals associated with bacteria living in biofilm consortia are seen as a prokaryotic paradigm for long‐distance electrical signaling in cellular communities (Prindle *et al*., 2015).

A recent study in phytoplankton (sized 2–200 μm) revealed that voltage‐gated Na^+^ channels are key to environmental sensing in the oceans (Helliwell *et al*., 2019). By contrast, diatoms in fresh water are suggested to use Ca^2+^ channels most likely in association with a Ca^2+^‐activated anion channel for depolarization and the firing of action potentials (for discussion, see Hedrich, 2019). Excitable animal systems build their electrical properties based on the microbial Na^+^ and Ca^2+^ channel ancestors. However, land plants only maintained the voltage‐dependent K^+^ channel but lost the archetypical Na^+^ and Ca^2+^ channels. Instead of voltage‐dependent Ca^2+^ channels, plants operate osmotically/mechanically and ligand‐gated Ca^2+^ channels.

What about action potentials (APs) in single cells of multicellular land plants? To polarize a membrane and fire an AP, only a small quantity of ions is needed. By contrast, osmotic effects (addressed later), such as turgor and volume changes, however, depend on mass flow of ions. Pollen tubes, root hairs, and stomatal guard cells belong to the latter category of systems (Blatt and Thiel described trains of action potential‐like membrane depolarizations in one Vicia faba guard cell exposed to as high as 100 μM auxin (IAA). Since this almost 30‐yr‐old report, neither Blatt and Thiel nor others have reported about such action potential‐like electrical activity in guard cells. This may mean that APs do not represent a major component in stomatal action. The AP is also not involved in synchronizing both guard cells of a stomatal complex because the single guard cell entities respond autonomously upon stimulation, that is, in response to closing signals.) (Blatt & Thiel, 1994). Among them, the guard cell is best understood with respect to the molecular nature and regulation of the ion channels, carriers, and pumps constituting the osmotic motor that drives cell expansion and movement of stomata formed by the guard cell pairs.

## Identifying the molecular makeup of the *Dionaea* action potential – a long and winding road

Besides providing a carnivorous plant system of proper size and well‐accessible physiology, the Venus flytrap served generations of electrophysiologists as the object of choice to study the biophysical features of an archetypical plant all‐or‐none action potential. For scientific generations this rather exotic plant was, however, not expected to get somewhere near a model system for electrical signaling. Instead, the ‘wonder weed’ *Arabidopsis thaliana* fulfills the criteria of a model system: small genome available at chromosomal resolution, single‐cell expression atlas, transformability, large set of ecotypes and broad mutant collection. Unfortunately, Arabidopsis lacks the all‐or‐none action potential, does not move, and cannot hunt and consume animals.

In the 2010s, techniques were advanced enough to potentially sequence the genome of any plant. When Hedrich and colleagues started the endeavor of sequencing *Dionaea's* genome, the Wuerzburg team was facing several surprises: the genome of *Dionaea* was found to be as large as the human genome (*c*. 3 Gbp, compared to 135 Mbp in Arabidopsis) and decorated with numerous transposons throughout. However, there were also lucky chances. The *de novo* assembly of the tissue‐specific transcriptomes took its time but was more straightforward at the end (Bemm *et al*., 2016). The genome of *Dionaea's* aquatic sister *Aldrovanda vesiculosa* that was sequenced in parallel is reasonably small; that is, 509 Mbp (Palfalvi *et al*., 2020). In 2018 the *Dionaea* team in Wuerzburg was contacted by the Mitsuyasu Hasebe lab to learn that they worked on the genome of the sundew *D*, as well a sister plant to *Dionaea* with a small genome size. Joint forces made it possible to make the draft genomes of all three Droseraceae relatives available in 2020 (Palfalvi *et al*., 2020). Another new ground was broken when Hiraku Suda in the Hasebe lab succeeded in introducing the genetically encoded calcium reporter GCaMP (synthetic fusion of green fluorescent protein (*G*FP), calmodulin (*CaM*), and M13, a peptide sequence from myosin light‐chain kinase) into the *Dionaea* genome (Suda *et al*., 2020). Using this tool, it was shown that touch‐induced cytoplasmic Ca^2+^ signals provoke trap closure.

**Fig. 1:**
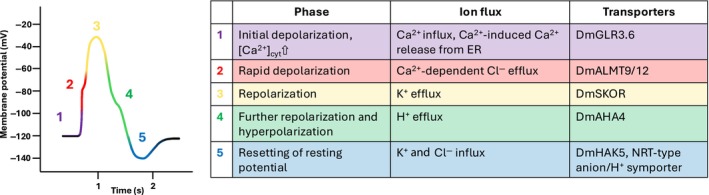
*Dionaea's* action potentials (AP) contains five distinct phases. (1) After an initial Ca^2+^ influx via DmGLR3.6, the Ca^2+^ signal is amplified by a Ca^2+^‐induced Ca^2+^ release (CICR) from the ER to an all‐or‐nothing cytosolic Ca^2+^ transient. (2) Once the Ca^2+^ level passes the threshold, Cl^−^ efflux via Ca^2+^‐dependent ALMT anion channels triggers an initial, rapid depolarization. Positive of −60 mV, depolarization‐dependent DmSKOR K^+^ channels, and DmAHA4 H^+^ pumps are activated and slow down the depolarization kinetics (first shoulder). At *c*. −20 mV, anion efflux and K^+^ efflux compensate each other so that the depolarization maximum is reached. The repolarization (3) sets in when [Ca^2+^]_cyt_ drops, the anion channel inactivates, and DmSKOR and DmAHA4 allow K^+^ and H^+^ release from the cell. (4) Negative of −60 mV DmSKOR inactivates, slowing the fast, initial repolarization (second shoulder). The delayed deactivation of the H^+^ pump AHA4 results in sustained H^+^ export, thus provoking a transient overshoot hyperpolarization. (5) During membrane hyperpolarization, DmAHA4 voltage inactivates and H^+^‐driven DmHAK5 and NRT type anion transporters reset the pre‐AP resting potential. Modified after Hedrich & Kreuzer (2023).

Studying the AP and GCaMP Ca^2+^ transient side‐by‐side, Scherzer *et al*. (2022b) documented that both signals propagate at the same speed. Following the application of the Ca^2+^ channel blocker La^3+^ or diethylether local anesthetics, touch‐induced calcium‐electrical signals and APs vanish with the same kinetics, indicating that the AP is Ca^2+^ dependent. To get hands on the molecular components of the AP, Scherzer *et al*. took a gain‐of‐function approach. In *Dionaea* only the upper part of the leaf forming a trap fires Ca^2+^‐APs upon stimulation, while this electrical signal is absent in juvenile, yet unopened traps (Scherzer *et al*., 2022a). When the transcriptomic profiles of the non‐excitable and excitable trap stages were analyzed, differentially expressed genes (DEGs) associated with ion transport attracted attention. Following the functional expression of these transporters in frog oocytes, inhibitor studies in the Venus flytrap and mathematical modeling could associate specific channels, pumps and carriers with the different phases of the AP (see model depicted in Fig. 1). In trap cells at rest (−120 mV), the AP is likely sparked by a voltage drop spilled over from excited trap segments and Ca^2+^ entry via glutamate receptor (GLR) type Ca^2+^ channels. This cytosolic Ca^2+^ spark may then be amplified to an all‐or‐none Ca^2+^ signal by an endoplasmic reticulum (ER)‐based calcium‐induced calcium release (CICR). In turn, voltage and Ca^2+^ activated Aluminum‐activated malate transporter (ALMT) type anion channels may open and shape the early depolarization phase (from −100 to −40 mV). When depolarization reaches the −40 mV threshold, a depolarization‐activated SKOR type K^+^ channel opens. This K^+^ release likely slows down the depolarization phase, thus reaching a tipping point at +20 mV. The repolarization phase could be initiated by the inactivation of the anion channel and depolarization activation of an AHA type H^+^ pump. When K^+^_out_ channels close at −40 mV, the H^+^ pump keeps on repolarizing the membrane potential which even causes a transient hyperpolarization overshoot relative to the initial resting potential. In the final phase of the AP, the H^+^ pump assumes its resting state and proton‐driven HAK5‐ and NRT‐type K^+^ and Cl^−^ symporters are thought to restore Cl^−^, K^+^, and H^+^ gradients and reset the pre‐AP membrane potential.

## The *Dionaea* trigger hair is a hapto‐electric switch

The *Dionaea* trigger hair is a touch‐sensory organ that translates mechanical energy into electrical energy. The upper part of the trigger hair serves as a lever that functions as a force amplifier. Micronewton forces generated by flies or other lightweight animals are translated into shearing stress that is picked up by a ring of mechanosensitive epidermal cells. Transcriptome analysis identified trigger hair‐specific DEGs, and some of these were localized to podium cells (Iosip *et al*., 2020). Among them, a mechanosensitive OSCA‐type Ca^2+^ channel and two MSL‐type anion channel homologs (addressed below) were spotted. When OSCA opens, the Ca^2+^ spark triggered by Ca^2+^ entering the podium cell very likely is amplified by an ER‐based CICR. To keep the trigger hair touch sensitive, Ca^2+^ must be cleared from the cytosol in the refractory period between subsequent stimulations. Among the DEGs, ACA and ECA Ca^2+^ ATPases are likely to play a role in resetting the pre‐stimulation Ca^2+^ background level. This touch‐induced Ca^2+^ transient can be monitored by GCaMP fluorescence imaging. In contrast to the cytosolic Ca^2+^ kinetics, the formation of touch APs in podium cells is more difficult to follow, because shearing forces will tend to displace intracellular electrodes in intact trigger hairs still attached to the trap. However, very recently simultaneous intracellular recordings of calcium and electrical signals led to the identification of a two‐step mechanical reception system in *Dionaea*'*s* sensory hairs, confirming DmMSL10/FLYC1 as the mechanosensor localized in the trigger hair podium (Suda *et al*., 2025).

Thus, trigger hair DEGs encoding the ALMT anion channel, the SKOR K^+^ channel, and an AHA H^+^ pump can only be assumed to play the same role in AP initiation in the trigger hair as in shaping the AP traveling the trap. A KAT type K^+^ uptake channel which was identified as trigger hair specific and localized in the podium of adult trigger hairs might be crucial for K^+^ loading and turgor formation of the mechanosensitive cells (Iosip *et al*., 2020). When the trigger hair K^+^ channel is blocked by Cs^+^ ions, high‐frequency AP induction is impaired. Unexpectedly, the ether‐sensitive glutamate receptor DmGLR3.6 was also found differentially upregulated in the fully excitable adult trigger hair (Scherzer *et al*., 2022a). Under ether anesthesia, touch still induced a Ca^2+^ rise in the trigger hair but blocked the propagation of the Ca^2+^ and electrical signal out of the trigger hair which is probably a result of ether sensitivity of DmGLR3.6.

How can one test models for the trigger hair initiation of the Ca^2+^‐AP and the propagation along the trap surface? Another ground was broken when very recently CRISPR/Cas9 gene editing was applied in *Dionaea*: the two trigger hair MSLs named FLYCATCHER (FLYC) 1 and 2, previously claimed to represent components of trigger hair mechanosensation, were mutated (Procko *et al*., 2021, 2023). The loss of these genes, however, did not affect trap closure in response to trigger hair bending significantly, but serves as proof of principle that model predictions for flytrap action can be critically tested (To carve out phenotypic differences between *flyc1/2* and WT plants, the Procko lab used sonication rather than touch. This way the entire trap becomes electrically excited and closes; that is, trap closure is induced in a trigger hair‐independent manner (cf. Scherzer *et al*., 2019 for experiments with the *Dionaea* trigger hair‐deficient mutant Basmati). The Toyota lab has recently developed a method to monitor touch APs from the intact trigger hair (Suda *et al*., 2025). In contrast to the Procko lab, the Toyota lab could document that the DmMSL10/FLYC1 anion channel is required to initiate the trigger hair AP, the Ca^2+^ wave, and finally trap closure.). Together, GCaMP gene expression and CRISPR/Cas9 gene editing will move *Dionaea* two steps forward towards a carnivorous and AP firing model plant.

## The trigger hair is a touch and heat sensor all in one

In the swamps of North and South Carolina, the home of the Venus flytrap, wildfires and prescribed burnings prevent the carnivorous plant population from being overgrown by grasses and shrubs. How can the Venus flytrap survive such fires? Huang & Hedrich (2023) have very recently documented that the flytrap operates a heat sensor. To simulate the heat waves in the forefront of a fire, the authors applied a heat ramp during which the temperature was raised from 20° to above 55°C. When the temperature reaches 37°C, a first AP gets fired followed by a second one when the 55°C threshold is passed. The trap closes after two heat‐triggered APs, which protects the trigger hairs. In contrast to a rapid heat wave, a slow rise in temperature, typical for a hot summer day in Carolina, does not provoke AP firing and trap closure. This reaction pattern tempted the authors to speculate that the heat sensor provides for an alarm system that recognizes heat waves in the forefront of wildfires.

Interestingly, such a heat response has not been described in *Arabidopsis*, although electrical and Ca^2+^ signals can be provoked by cold, touch, and high voltage stimulation there. When exposed to the latter stimuli, flytraps fire the same archetypical all‐or‐none AP as that triggered by heat. When successively stimulated towards the first temperature threshold of 37°C only, the heat sensor is subject to inactivation characterized by a refractory period of 3 min. During the refractory period, cold, touch, and high voltage stimulation still trigger AP firing. Given that the heat‐induced Ca^2+^ signal, as well as that provoked by touch, emerge from the trigger hair podium, one cannot exclude that mechanosensitive OSCAs might be activated by heat‐induced structural rearrangements within the channel protein.

In contrast to the animal field, plant receptors initiating Ca^2+^ signals in response to heat and cold stimuli remain unknown. In this context, it is astonishing, that the many excellent studies and screens to identify the cold sensor in *Arabidopsis* have not spotted a cold‐activated Ca^2+^ channel yet. Maybe optimized very sensitive screens with the good, old Ca^2+^ reporter aequorin (Knight *et al*., 1991) that helped to identify OSCA1, HPCA1 or GIPC sphingolipids as osmotic sensors and sensors for H_2_O_2_ or Na^+^, respectively, will also lead to the discovery of Ca^2+^ channels that are activated by thermal energy in the not‐so‐far future (Jiang *et al*., 2019; Wu *et al*., 2020; Pei *et al*., 2022).

## How much information does the electrical signal carry and how is it read?

In a previous study, it was shown that the flytrap can make sense of the number of action potentials emitted by the trigger hair hapto‐electrics (Böhm *et al*., 2016). In response to one AP, there is no action, but the first AP is memorized until another electrical signal is received by the trap lobe motor cells within the following 30 s. Closure upon the elicitation of two APs largely reduces the risk of a false response and increases the probability that the release of visco‐elastic energy stored in the open trap will lead to the capture of a living, mobile animal. APs number 3–5 provoked by the encaged prey sustainedly striking the trigger hairs when trying to break out of the green prison, induce touch hormone JA signaling and synthesis. JA stimulates the gland‐based endocrine system, lytic enzymes are released, prey is being processed, transporters are synthesized, and nutrients are taken up from the animal food stock. The hunting cycle of the flytrap can be subdivided into three branches: (1) prey capture by snap closure after two APs, (2) biosynthesis of JA, and (3) JA‐dependent prey consumption after more than three APs. Rapid trap closure is Ca^2+^ dependent, but JA independent. JA biosynthesis has been shown to depend on transient cytoplasmic Ca^2+^ elevation. The fact that the early steps of JA biosynthesis take place in the chloroplast, raises the question of how cytoplasmic Ca^2+^ signals are translated into chloroplast JA biosynthesis.

**Fig. 2:**
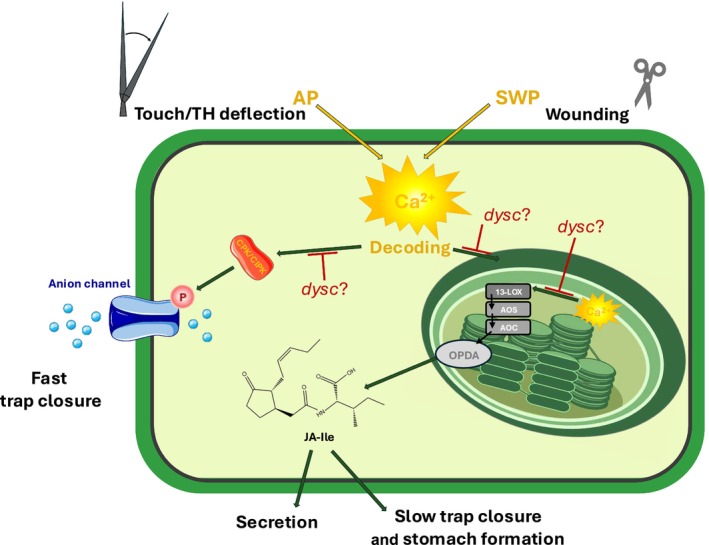
Various triggers (e.g. touch, wounding) induce different Ca^2+^ signatures (and electrical signals such as APs and slow wave potentials (SWPs)) that are very likely processed by diverse Ca^2+^ decoders. The *Dionaea* mutant *dysc* is very likely impaired in addressing Ca^2+^ signaling elements leading to the activation of anion channels and thus fast, JA‐independent, trap closure. Moreover, *dysc* is also unable to properly transmit Ca^2+^ waves into the initiation of JA biosynthesis and thus the progression of its hunting cycle. Modified after Iosip *et al*. (2023).

The Wuerzburg *Dionaea* team had another lucky chance to obtain the *Dionaea* mutant ‘ERROR’ from the horticulturist Matthias Maier (https://www.green‐jaws.com). The mutant was renamed DYSCALCULIA (*dysc*) to better fit its malfunction: *dysc* is firing normal APs upon touch but does not snap close in response to two APs and is unable to translate the higher‐order AP counts into proper JA biosynthesis (Iosip *et al*., 2023). In other words, *dysc* is impaired in decoding the cytosolic Ca^2+^ signals to stimulate JA production. When comparing DEGs between WT and *dysc* in the ground state and in response to mechanostimulation, Iosip *et al*. identified target genes of transcription factors or calcium decoders already deregulated (i.e. up‐ or downregulated) in *dysc* ground state. Among them we also spotted an ortholog of the 13‐lipoxygenase AtLOX3, known to produce JA precursor molecules in Arabidopsis (Caldelari *et al*., 2011), as downregulated in *dysc*, which might explain the lower level of JA induction upon wounding in the mutant (Fig. 2).

*Arabidopsis* responds to a single touch by a single AP that lasts 10–20 s (Degli Agosti, 2014). In contrast to touch, wounding the model plant causes a more complex electrical response called variation or slow wave potential (SWP), since its shape and amplitude differ from one experiment to another. The electrical response to touch and wounding is always accompanied by a Ca^2+^ wave (Nguyen *et al*., 2018; Toyota *et al*., 2018; Hagihara *et al*., 2022). Thus, in noncarnivorous plants as well, cytoplasmic Ca^2+^ signals seem to provide an integral part of the local and systemic electrical response. In addition to a rise in calcium, JA is central in the response to touch and wounding in Arabidopsis (Farmer *et al*., 2020).

One of the major questions in the *Arabidopsis* mechanical stress response is how to distinguish between touch and wounding. In other words, is touch already a kind of wounding? While the *Arabidopsis* trichome had already been proposed to function as a mechanosensory switch initiating apoplastic alkalinization and Ca^2+^ oscillation in surrounding cells in response to touch stimulation (Zhou *et al*., 2017), falling raindrops had not been associated with mechanical stress before Matsumura *et al*. (2022) performed a set of experiments that received much attention. The authors could show that ordinary trichomes of *Arabidopsis* are sensory units that pick up the shearing stress imposed by falling raindrops and translate mechanical energy into a Ca^2+^ signal (chemical energy). The genetically encoded calcium indicator GCaMP was used to visualize calcium waves originating from trichomes mechanically stimulated by falling raindrops. After a raindrop struck a trichome, a Ca^2+^ wave concentrically expanded from the site of mechano‐sensation toward the neighboring cells of the leaf sector. Astonishingly, raindrops falling onto Arabidopsis leaves resulted in the induction of *c*. 1000 genes already 15 min after stimulation onset. Transcriptome analysis revealed that this treatment activates mechanosensitive genes such as TCH2 and TCH4 and typical JA genes like JAZ1 through the CALMODULIN‐BINDING TRANSCRIPTION ACTIVATOR 3 (CAMTA3) and mitogen‐activated protein kinases (cf. flytrap gene expression after trigger hair stimulation described below).

Like falling raindrops, mechanostimulation of a single trichome with a wire triggered an intercellular calcium wave. Quantitative Ca^2+^ and glutamate imaging was used to demonstrate that traveling calcium waves in response to touch and wounding are mediated by the flow of glutamate (Bellandi *et al*., 2022). The authors proposed that mechanical stimuli trigger the release of glutamate that diffuses locally through the apoplast and activates the calcium‐permeable channel GLUTAMATE RECEPTOR‐LIKE channel GLR3.3. Later, it was reported that, *in vivo*, glutamate‐dependent activation of the AtGLR3.3 channel requires a functional extracellular glutamate‐binding domain of the receptor Ca^2+^ channel. Combining imaging and genetics, it was shown that leaf mechanical stress induces the systemic increase of L‐glutamate in the cells' external compartment and in turn cytosolic Ca^2+^ elevation (Grenzi *et al*., 2023).

Wounding‐induced, GLR‐based electrical impulses are not only variable but often quite complex in nature. Besides the fast initial depolarization phase no underlying pattern can be determined. Such a complex signal can form when primary mechanical–electrical cues and chemically induced secondary electrical events superimpose. The situation could complicate further when the different components use different cellular networks or even temporally different directions and meet again at contact points. Glutamate and ATP are released by injured cells and bind to the extracellular binding sites of their respective receptors, GLRs and purinergic receptors of the P2K family such as DORN1 (Cho *et al*., 2017; Grenzi *et al*., 2022). Upon ligand binding, GLR‐type receptor channels open right away to mediate Ca^2+^ influx and membrane depolarization. ATP receptors of the DORN1 type are proposed to activate downstream signaling cascades including Ca^2+^ and reactive oxygen species (ROS) after perception of extracellular ATP (Choi *et al*., 2014; Cho *et al*., 2017). Given that glutamate and ATP represent elicitors of different nature, chances are high that other cellular compounds leaking out of wounded cells trigger chemical‐electrical signals as well.

Recently, the nature of a so‐called Ricca's factor has been identified by the Ted Farmer lab (Gao *et al*., 2023). Leaf‐feeding insects trigger defense‐inducing electrical signals (slow wave potentials) that are elicited by Ricca's factors traveling the primary veins. In *Arabidopsis*, such a Ricca factor was identified as thioglucosidase (TGG) proteins, which are able to break down aliphatic glucosinolates, thus releasing reactive aglycones that are necessary to trigger SWPs, Ca^2+^ transients and JA signaling (Gao *et al*., 2023). Propagation of Ca^2+^ electrical waves from insect feeding sites is absent in glr3.3/3.6 mutants and strongly attenuated in tgg1/2 mutants (Xue *et al*., 2022; Gao *et al*., 2023). Applying recombinant TGG1 into the xylem of the tgg1/2 double mutant elicited wild‐type‐like membrane depolarization and Ca^2+^ transients (Gao *et al*., 2023). These findings raise questions about how xylem mobile Ricca's factors can feed forward phloem traveling Ca^2+^ electrical signals. Do glucosinolates impact glutamate release or modulate GLR function?

Like raindrops falling on Arabidopsis trichomes, insects visiting the Venus flytrap and colliding with the trigger hairs (multicellular trichomes) are not regarded as mechanical stress or wounding. However, bending *Dionaea's* trigger hair by only 3° provokes a single Ca^2+^‐AP (Scherzer *et al*., 2019). Touch‐induced calcium‐electrical signals travel the entire trap surface but not the cells of the rim and the spine‐like outgrowths. Wounding the trap, however, provokes a more complex calcium‐electric signal based on instantaneous short sequences of archetypical Ca^2+^ APs followed by a secondary slow Ca^2+^ wave that also extends to the rim and spines. When comparing *Dionaea's* response to touch and wounding, JA responses were found to differ in amplitude and kinetics. While JA levels quickly rise within 15 min after mechanostimulation, an increase in JA is only observed from 1 to 3 h after wounding (Iosip *et al*., 2023). One may therefore assume that the slow Ca^2+^ wave triggers the wounding‐induced gene expression as well as increased JA levels.

In tomato plants, herbivory‐/wounding‐induced Ca^2+^ signals trigger JA biosynthesis via Calmodulin2 (CaM2) and Ethylene Response Factor 16 (ERF016) (Hu *et al*., 2022). ERF016/ORA47 is also transcriptionally induced in *Dionaea* WT plants in response to wounding, whereas this induction is completely absent in the *dysc* mutant mentioned earlier, which is impaired in properly transmitting the Ca^2+^ signals into JA biosynthesis. In Arabidopsis, ORA47 is regulated by the Calmodulin‐binding transcription activator CAMTA3 (Zeng *et al*., 2022). Interestingly, CAMTA3 has been lost in all Droseracea sequenced so far. The remaining three CAMTAS in *Dionaea* have very low expression in both WT and *dysc* and are not transcriptionally regulated after any of the stimuli (Iosip *et al*., 2023). Therefore, ERF016/ORA47 might be the target of a different Ca^2+^‐binding regulator; that is, *Dionaea* seems to thread a different path to address JA biosynthesis, which will be the subject of future analyses.

As mentioned earlier, the *dysc* mutant fails to properly decode the Ca^2+^ signals evoked by different stimuli, thus resulting in reduced JA levels in response to mechanostimulation and complete suppression of wounding‐induced JA biosynthesis. *Dysc's* JA phenotype is specific for the trap, since wounding the petiole induced WT‐like JA production in *dysc* as well, which raises questions about how this Ca^2+^/JA phenotype is restricted to *Dionaea's* capture organ.

## The monkeyflower *Mimulus* as a new model for tissue excitability?

Monkeyflowers (*Mimulus*) have long been recognized as a classic ecological and evolutionary model system that holds great promise for studying developmental genetics. Recent progress has been made in understanding the molecular basis of floral trait divergence underlying pollinator selectivity (Nelson *et al*., 2021). Why is this mentioned in an essay about ionic movements in plants? The stigmata of some *Scrophulariaceae* species are touch sensitive and respond to pressure by closing, most likely to prevent double fertilization or self‐pollination within a flower during a pollinator's visit. The bilobed, papillose stigma in hummingbird‐pollinated *Mimulus aurantiacus*, for example, closes within seconds after the tactile stimulus. Stigmata start to reopen within 2–4 h if only touch but no pollen is applied. By contrast, stigmata that received pollen, meaning that the touch signal is followed by a chemical one, stay closed and initiate the next steps of fertilization (Fetscher & Kohn, 1999).

This behavior is reminiscent of the ion motive force‐powered movements in the Venus flytrap and that of stomatal guard cells: All three systems can sense mechanical forces. What are the similarities and the differences? The open *Dionaea* trap represents a metastable, prestressed system (Forterre *et al*., 2005; Sachse *et al*., 2020). Upon touch perception, an AP travels the whole trap, finally initiating the release of viscoelastic energy, abrupt curvature inversion of the trap lobes (i.e. snap buckling) and thus rapid trap closure. Snap buckling is very likely enabled by simultaneous expansion of outer cell layers and shrinkage of cells located at the inner epidermis close to the midrib (Sachse *et al*., 2020), thus representing a turgor‐driven plant movement, although water fluxes have not yet been reported in detail. However, the ion channels and transporters underlying AP generation and mechanoperception have already been suggested (Iosip *et al*., 2020; Procko *et al*., 2021; Scherzer *et al*., 2022a; Hedrich & Kreuzer, 2023; Suda *et al*., 2025).

In *Mimulus*, the sequence of events is similar: here, touch perception and elicitation of an action potential also represent the closing signals leading to the rapid movement of the stigma lobes. If there is no further signal, the stigma will reopen, whereas sustained stigma closure in the presence of pollen is under control of self‐pollination receptor biology. The molecular players responsible for *Mimulus* mechanoperception and AP generation have yet to be identified. However, the MscS‐like gene JUE1 has been recently described in *Torenia fournieri*, a species closely related to *Mimulus* (Zhou *et al*., 2025). Mutant plants lacking JUE1 are impaired in spreading the touch‐induced [Ca^2+^]_cyt_ wave and fail to close their stigma in response to mechanostimulation. It was therefore proposed that JUE1, alike its orthologs in *Arabidopsis* and *Dionaea*, MSL10 and FLYC1, might release Cl^−^ and thus initiate a [Ca^2+^]_cyt_ wave via depolarization‐activated Ca^2+^ channels. In the future, combined monitoring of Ca^2+^ and and APs will help specifying JUE1's function in stigma hapto‐electrical signaling and movement.

While the movement of stomata is much slower, it is still driven by turgor changes powered by a hydrodynamic system involving the guard cells themselves as well as neighboring cells. There is no AP generation, but three types of mechanosensitive, stretch‐activated ion channels contribute to guard cell volume regulation (Cosgrove & Hedrich, 1991). Closure of stomata open in the light is initiated by darkness, low air humidity, abscisic acid, and microbial factors such as chitin and flagellin and the molecular identity of the underlying ion channels has been described in great detail (Kollist *et al*., 2014).

## Can ion channel‐based optogenetics provide a powerful tool to better understand calcium‐electrical signaling in plants?

Like the Venus flytrap, touch‐sensitive excitable *M. pudica* plants can fire an all‐or‐nothing AP (Volkov *et al*., 2010). In this system, a Ca^2+^ wave propagates along the same route and at the same speed as the AP (Hagihara *et al*., 2022). This documents that the calcium and the electrical signal are two sides of the same coin. A change in membrane polarization of animal cells can activate voltage‐dependent phosphatases and kinases translating electrical into phospho‐chemical energy. In contrast to the animal system, conversion of voltage signals into (de)phosphorylation of membrane‐associated proteins has not been reported in plants yet. In all systems investigated so far, the action potential in excitable plants as well as stimulus‐induced electrical signals in nonexcitable plants such as *Arabidopsis* originate from or are closely associated with a transient rise in cytoplasmic Ca^2+^ (Matsumura *et al*., 2022).

Is the plant reading the electrical or the Ca^2+^ signal or a combination of both pieces of information? To answer the question of which energy form a plant ‘counts’, new tools such as noninvasive optogenetics will be useful. Channel‐forming rhodopsins (CHRs) of algae phytoplankton are widely used in neurobiology because they operate as light‐activated membrane‐localized ion gates with different selectivities (Hegemann & Nagel, 2013; Emiliani *et al*., 2022; Duan *et al*., 2025; Hedrich & Gilliham, 2025). In plant membrane‐based signaling, the cations Ca^2+^ and H^+^ play a dominant role, whereas K^+^, which is found in the 100 mM range in plant cells, rather serves housekeeping functions. Plant cells are surrounded by a kind of pond water, a moiety of low ionic and osmotic strength. In this scenario, the plasma membrane K^+^ and anion gradients are directed outward while that of Ca^2+^ and H^+^ is directed inward. In contrast to nerve and muscle cells, anion channels have been shown to be major drivers of plant signal‐specific membrane depolarization. Ion channels of the SLAC/SLAH type are activated by CDP and CBL/CIP kinases (Maierhofer *et al*., 2014; Hedrich & Geiger, 2017; Huang *et al*., 2023a). These Ca^2+^ kinases convert the chemical energy of the cytoplasmic Ca^2+^ rise into the transfer of a high‐energy phosphate from ATP to target sites on the anion channel protein. As a result of high‐energy phosphotransfer, SLAC/SLAH anion channels gate open. Following the transformation of the CHR gene acr1 into plants, it was shown that light activation of the anion‐selective microbial channel depolarizes the guard cell membrane and causes stomatal closure (Huang *et al*., 2021). This indicates that membrane depolarization is essential and sufficient for stomatal closure and that Ca^2+^ kinase activation of guard cell SLAC1 and SLAH3 represents a crucial upstream step.

How can we learn more about the information carried by the Ca^2+^ signal? Why not use Ca^2+^ permeable CHRs to trigger a light‐induced cytosolic Ca^2+^‐transient to activate guard cell intrinsic SLAC1 and SLAH3 anion channels in the next step? Noninvasive remote controlling of cytoplasmic Ca^2+^ via Ca^2+^ channel rhodopsins will help answer if and how plants can read the amplitude of a Ca^2+^ transient, the number of Ca^2+^ waves and/or their frequency (Huang *et al*., 2023b, 2024). Calcium‐induced stomatal closure takes place in the 10 min range and is thus unlikely to involve transcriptional regulation. In most other cell types, Ca^2+^‐dependent signaling pathways manifest in an hour timescale and involve Ca^2+^‐regulated transcription. Taking advantage of the depolarizing anion channel ACR1 and the Ca^2+^ channel XXM as well as hormone, metabolite and RNA profiling, the information of the pure voltage and Ca^2+^ signal can be deconstructed (Ding *et al*., 2024; Song *et al*., 2025).

## Future directions

Using different channelrhodopsin variants in optogenetic studies, we will not only be able to analyze rapid plant movements in detail but also Ca^2+^‐dependent processes underlying plant growth and development. When imposing defined cytosolic Ca^2+^ signatures via XXM2.0, short cytosolic Ca^2+^ transients were sufficient to attenuate auxin‐induced membrane depolarization, transcriptional activation and root growth in Arabidopsis seedlings. These effects were shown to be dose‐dependent and reversible, thus underscoring the dynamic and tunable nature of auxin sensitivity in plant roots (Song *et al*., 2025). In a related study, cytosolic Ca^2+^ elevation turned out to be both necessary and sufficient for rapid inhibition of root growth in Arabidopsis. Interestingly, the rise in cytoplasmic Ca^2+^ by activation of ER‐targeted XXM2.0 caused root growth inhibition like plasma membrane‐localized XXM2.0. The latter finding supports the notion that the ER operates as a store that is home to calcium‐induced calcium release (Scherzer *et al*., 2022b; Huang *et al*., 2024). Among other techniques, optogenetic manipulation of [Ca^2+^]_cyt_ revealed that diverse physiological and environmental cues all converge on pathways that elevate cytosolic Ca^2+^, leading to rapid root growth inhibition (Randuch *et al*., 2025).

Both papers provide evidence placing cytosolic Ca^2+^ at the center of rapid root growth regulation in Arabidopsis, integrating multiple hormonal and stress signals by acting as either a master signal or a reversible gate for hormonal sensitivity. Optogenetic control of Ca^2+^ concentration therefore offers a powerful tool to dissect the crosstalk between phytohormones and the second messenger with unprecedented temporal resolution.

## Competing interests

None declared.

## Author contributions

RH and IK conceptualized the study, wrote the original draft, and reviewed and edited the manuscript.

## Disclaimer

The New Phytologist Foundation remains neutral with regard to jurisdictional claims in maps and in any institutional affiliations.
